# Resting-state functional connectivity and socioemotional processes in male perpetrators of intimate partner violence against women

**DOI:** 10.1038/s41598-022-14181-2

**Published:** 2022-06-16

**Authors:** Sofia Amaoui, Cristina Martín-Pérez, Agar Marín-Morales, Natalia Bueso-Izquierdo, María Ángeles García-León, Miguel Pérez-García, Juan Verdejo-Román

**Affiliations:** 1 The Mind, Brain and Behavior Research Center (CIMCYC), Granada, Spain; 2 Department of Personality, Assessment and Psychological Treatment, University of Granada, Granada, Spain; 3 Faculty of Education, Universidad Internacional de la Rioja, Logroño, Spain; 4 Department of Clinical and Experimental Psychology, University of Huelva, Huelva, Spain; 5 Faculty of Education and Psychology, Department of Psychology and Anthropology, University of Extremadura, Badajoz, Spain; 6 FIDMAG Sisters Hospitallers Research Foundation, Barcelona, Spain; 7 Centro de Investigación Biomédica en Red de Salud Mental, Instituto de Salud Carlos III, Barcelona, Spain; 8 Department of Experimental Psychology, Cognitive Processes and Speech Therapy, Complutense University of Madrid, Madrid, Spain; 9 Laboratory of Cognitive and Computational Neuroscience (UCM-UPM), Centre for Biomedical Technology (CTB), Madrid, Spain

**Keywords:** Neuroscience, Biomarkers

## Abstract

Intimate partner violence against women (IPVAW) is a serious and overwhelming public concern. Neuroimaging techniques have provided insights into the brain mechanisms underlying IPVAW perpetration. The purpose of this study is to examine the resting-state functional connectivity (rsFC) involving the process of social decision-making of male perpetrators. Twenty-six male perpetrators convicted for an IPVAW crime were compared to 29 men convicted for crimes other than IPVAW (other offenders) and 29 men with no criminal records (non-offenders) using a seed-based approach. Seeds were located in areas involved in reflective (prefrontal), impulsive (amygdala and striatum) and interoceptive (insula) processing. Then, as an exploratory analysis, the connectivity networks on male perpetrators were correlated with measures of executive functions and socioemotional self-report measures. Male perpetrators in comparison to other offenders and non-offenders, presented higher rsFC between prefrontal, limbic, brainstem, temporal and basal ganglia areas. Also male perpetrators showed higher rsFC between insula, default mode network and basal ganglia, while lower rsFC was found between prefrontal and motor areas and between amygdala, occipital and parietal areas. Exploratory correlations suggest that the specific rsFC in male perpetrators might be more related to socioemotional processes than to executive functions. These results showed that male perpetrators present a specific rsFC in brain systems that are essential for an adaptive social decision-making.

## Introduction

Intimate partner violence against women (IPVAW) has become a priority social issue and a major public health concern due to the alarming statistics and severity^1^. Previous literature has highlighted not only the high prevalence of violence against women globally, but also its adverse physical, psychological, social and economic harm^2–4^. Due to its complex and multicausal nature^5^, new approaches have been developed to achieve a better comprehension of this specific violence^6,7^. Neuroscientific advances have offered the opportunity to study brain functioning in male perpetrators as a new key component for IPVAW research^8^.

Over the last decade, research in IPVAW has focused on studying different processes such as social decision-making in order to understand why male perpetrators engage in violence against their female intimate partner^9^. Social decision-making is defined as the process by which people make decisions that affect others as well as themselves^10^, which is crucial for adaptive social interactions^11^. According to the Triadic Reflective–Impulsive–Interoceptive Awareness Model^12^, decision-making is sustained on the basis of three differentiated but dependent systems. First, the Reflective prefrontal brain system which is involved in executive functions, cognitive and emotional control^13^. Second, the Impulsive amygdala-striatal brain system which coordinates automatic and impulsive behaviors^14^. Finally, the Interoceptive-awareness insular brain system which is implicated in perceiving, processing and representing afferent internal signals^15^. Alterations in these processes may lead to maladaptive and violent behaviors^16^.

Previous literature reveals that male perpetrators present different activation in brain areas within these three systems during emotional processing and emotional regulation fMRI tasks in comparison to other offenders and non-offenders. More concretely, a study of Lee et al.^17,18^ demonstrated less activation of prefrontal areas and higher activation of the limbic system (amygdala) and insula in male perpetrators compared to controls in response to aggressive stimuli. Another study^19^ demonstrated similar results in male perpetrators in comparison to other convicted men. Finally, a recent study^20^ showed that male perpetrators present specific prefrontal and amygdalar activation during different emotional regulation processes in comparison to non-offenders and other offenders. Interestingly, these few neuroimaging results have been obtained studying brain activation during fMRI-tasks, and no study has explored brain functioning under resting-state. Over the last few years, a new research line interested in studying whether the resting-state functional connectivity (rsFC) could be a predictor of violent proneness has emerged^21^. In this sense, a recent study^22^ has shown that violent men (including male perpetrators) showed different connectivity between prefrontal, amygdalar and insular areas in comparison to non-offenders during a resting-state scan and proposes that different types of violence might be associated to different functional connectivity^16^. All these studies suggest a different connectivity between prefrontal areas (reflective brain system) and amygdalar and insular areas (impulsive and interoceptive brain systems), underlying an inadequate top-down regulation in male perpetrators.

Therefore, the purpose of this research is to study, for the first time, the resting-state functional connectivity of the brain systems involved in social decision-making^12^ in male perpetrators and compare it to two groups: men with no criminal records and men convicted for crimes unrelated to IPVAW^19^. Moreover, as an exploratory aim, we examined the possible association between the specific functional connectivity of male perpetrators and the executive functioning (i.e.: updating process, inhibition, decision-making and cognitive flexibility), and the socio-emotional processes (i.e.: empathy, emotion recognition, emotion regulation, distorted thoughts about women and violence and impulsivity) previously found altered in this population.

To date, there are no previous studies exploring the rsFC in male perpetrators and given the novelty of this line of research, we did not make a priori hypotheses about the specific connections that would be implicated nor about the directionality of effects, however, based on the resting-state literature in violent populations^21,22^ and the studies of brain activation in male perpetrators^9,17,19^, we hypothesized that male perpetrators would present a different resting-state functional connectivity in comparison to non-offenders and other offenders, between prefrontal areas (reflective system) and amygdala-striatal and insular areas (impulsive and interoceptive system). As an exploratory hypothesis, we hypothesized that specific functional connectivity in male perpetrators would be associated with executive functions and socioemotional processes.

## Results

### Demographic data

Male perpetrators (MPG; N = 26), men convicted of crimes other than IPVAW (OOG; N = 29) and men with no criminal records (NOG; N = 29) were compared in relation to sociodemographic variables. There were no between-group differences in age or education level. As expected, differences were found in the severity scale of the Conflict Tactic Scale^23^, demonstrating that MPG reported higher scores in comparison to OOG and NOG. This scale evaluates the frequency and intensity of the violence toward an intimate partner. Sociodemographic and crime characterization data are presented in Table 1.

**Table 1:** Sociodemographic and crime characteristics of MPG, OOG and NOG.

Variables	MPG (n = 26)	OOG (n = 29)	NOG (n = 29)	*F*/*χ*^2^	*p*-value
Age (years)	41.19 (9.71)	38.97 (11.05)	38.28 (8.54)	0.66	0.51
Years of education	9.19 (4.30)	9.55 (3.58)	9.86 (2.44)	0.251	0.77
Severity (CTS-2)	4.27 (6.27)	0.24 (0.51)	0.31 (0.93)	11.43	< 0.0001
Drug severity	1.11 (0.40)	1.09 (0.36)	0.91 (0.33)	2.61	0.08
**Loss consciousness**					
Yes (< 30 min)	3.8% (1)	3.4% (1)	0% (0)	2.658	0.954
Yes (< 15 min)	19.3% (5)	13.7% (4)	20.6% (6)		
No	77% (20)	82.7% (24)	79.3% (23)		
**Indirect violence during childhood**				1.177	0.55
Yes	20% (5)	20.7% (6)	31%(9)		
No	80% (20)	79.3% (23)	69% (20)		
**Direct violence during childhood**				1.580	0.45
Yes	12% (3)	24.1% (7)	24.1% (7)		
No	88% (22)	75.9% (22)	75.9% (22)		
**Type of crime**					
	PV = 57.7% (15)	SCF = 10.3% (3)			
	PPV = 42.3% (11)	DD = 17.24% (5)			
		GAR = 24.1% (7)			
		DT = 34.5% (10)			
		AA = 3.4% (1)			
		UM = 10.3% (3)			

Except for type of crime, violence during childhood and loss of consciousness, all values are mean (± SD). *MPG* male perpetrators group, *OOG* other offenders group, *NOG* non-offenders group, *CTS-2* Conflict Tactic Scale-2, *Indirect violence* witnessing violence during childhood, *Direct violence* experiencing violence during childhood, *PV* psychological violence, *PPV* physical and psychological violence, *SCF* scams or crimes of forgery, *DD* dangerous driving, *GAR* Grave assault/robbery, *DT* drug trafficking, *AA* attack on authority, *UM* unspecified misdemeanor (lost answers).

### Seed-based functional connectivity results

Based on the main objective of the study, the results were organized as follows:

#### Male perpetrators group vs non-offenders group

**Figure 1:**
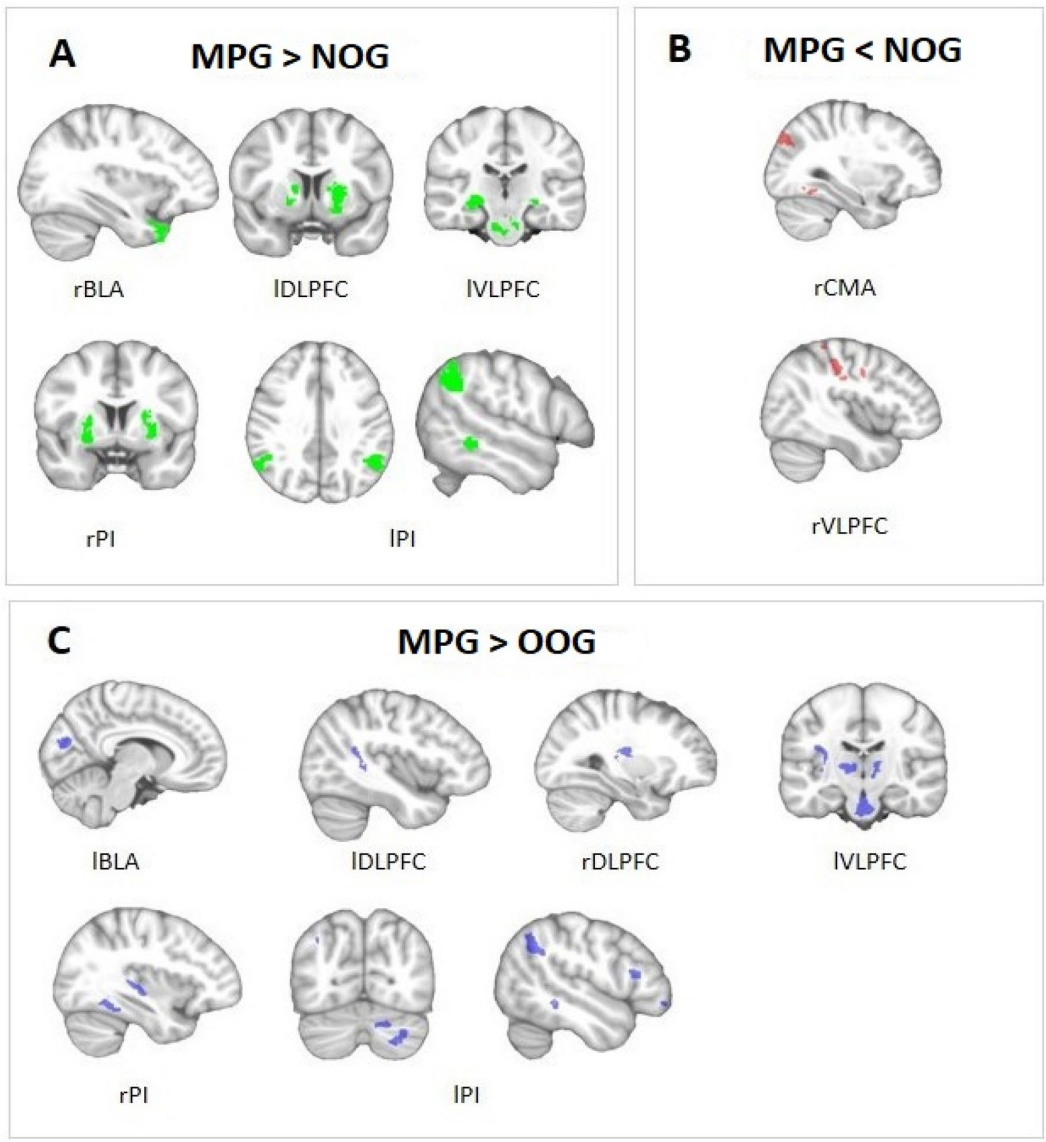
Significant group differences in seed-based analysis. Each brain image represents the correlations with the seed written below. *MPG* male perpetrators group, *OOG* other offenders group, *NOG* non-offenders group. (**A**) MPG demonstrated higher functional connectivity than NOG between rBLA (right basolateral amygdala) and temporal pole, between lVLPFC (left ventrolateral prefrontal cortex) and brainstem, middle temporal area and bilateral hippocampus, between lDLPFC (left dorsolateral prefrontal cortex) and putamen-caudate, between rPI (right posterior insula) and putamen and finally between lPI (left posterior insula) and bilateral angular gyrus and middle temporal area. (**B**) MPG demonstrated lower functional connectivity than NOG between rCMA (right centromedial amygdala), intraparietal, fusiform gyrus, and occipital area, and between rVLPFC (right ventrolateral prefrontal cortex) and sensorimotor, premotor, intraparietal and occipital areas. (**C**) MPG showed higher functional connectivity in comparison to OOG between lBLA (left basolateral amygdala) and visual-cuneus, between lVLPFC (left ventrolateral prefrontal cortex) and brainstem, insular cortex and bilateral thalamus, between rDLPFC (right dorsolateral prefrontal cortex) and sensorimotor, between lDLPFC (left dorsolateral prefrontal cortex) and middle temporal gyrus, between rPI (right posterior insula) and fusiform gyrus and Heschl Gyrus, and finally between lPI (left posterior insula) and angular gyrus, middle temporal, putamen, dorsolateral cortex, cerebellum and inferior frontal area.

Regarding the impulsive system, MPG showed higher connectivity between the rBLA seed and temporal pole but lower connectivity between the rCMA seed intraparietal area, fusiform gyrus and occipital area. Moreover, in the reflective system, MPG showed increased functional connectivity between lVLPFC seed and brainstem, bilateral hippocampus and middle temporal area, and between lDLPFC seed and bilateral putamen-caudate. However, lower functional connectivity was found between rVLPFC seed and sensorimotor area, premotor area, intraparietal sulcus and occipital area. Finally, within the interoceptive system, MPG demonstrated higher connectivity between PI seed and bilateral putamen and between lPI seed and bilateral angular gyrus and middle temporal area (Table 2, Fig. 1).

**Table 2:** Significant differences between MPG and NOG in seed-based functional connectivity.

Seed	Brain region	x	y	z	Ke	Peak t value	Seed’s system
**MPG > NOG**							
rBLA	Temporal pole	− 34	20	− 34	111	4.76	Impulsive
lVLPFC	Brainstem	12	− 22	− 30	234	4.49	Reflective
	Middle Temporal area	− 48	− 36	− 8	167	4.28	
	Hippocampus	− 18	− 40	0	99	4.38	
	Hippocampus	26	− 24	− 4	116	4.58	
lDLPFC	Putamen-caudate	− 16	12	6	592	4.60	Reflective
	Putamen-caudate	8	0	− 4	298	4.97	
rPI	Putamen	− 30	0	0	328	4.67	Interoceptive
	Putamen	24	4	− 6	164	4.17	
lPI	Angular Gyrus	− 50	− 54	38	470	4.73	Interoceptive
	Angular Gyrus	62	− 56	36	212	4.62	
	Middle temporal area	− 56	− 40	− 8	140	4.68	
**MPG < NOG**							
rCMA	Intraparietal	30	− 80	40	160	5.29	Impulsive
	Fusiform gyrus	36	− 56	− 14	116	5.21	
	Occipital area	16	− 46	− 12	112	4.85	
rVLPFC	Sensorimotor area	40	− 22	34	150	4.13	Reflective
	Premotor area	44	− 4	38	148	5.40	
	Intraparietal sulcus	34	− 48	64	117	4.49	
	Occipital area	− 38	− 86	18	128	4.60	

Coordinates (x, y, z) are given in Montreal Neurological Institute atlas space (MNI). *Ke* cluster size in voxels; All the results have exceeded the minimum threshold p < 0.001 and number of voxels for each seed, *Seed’s system* system to which each seed corresponds according to the Triadic reflective–impulsive–interoceptive awareness model, *MPG* male perpetrators group, *NOG* non-offenders group, *rBLA* right basolateral amygdala, *lVLPFC* left ventrolateral prefrontal cortex, *lDLPFC* left dorsolateral prefrontal cortex, *rPI* right posterior insula, *lPI* left posterior insula, *rCMA* right centromedial amygdala, *rVLPFC* right ventrolateral prefrontal cortex.

#### Male perpetrators group vs other offenders group

Regarding the impulsive system, MPG demonstrated higher functional connectivity between the lBLA seed and visual-cuneus. Moreover, in the reflective system, MPG showed higher functional connectivity between the lVLPFC seed and brainstem, bilateral thalamus and insular cortex, between rDLPFC seed and sensorimotor area and between lDLPFC seed and middle temporal gyrus in comparison to OOG. Finally, within the interoceptive system, MPG demonstrated higher connectivity between rPI seed and fusiform gyrus and Heschl gyrus, and between lPI seed and left angular gyrus, middle temporal area, left putamen, cerebellum, dorsolateral cortex and inferior frontal area. MPG did not demonstrate lower functional connectivity than the OOG in any of the selected seeds (Table 3, Fig. 1).

**Table 3:** Significant differences between MPG and OOG in seed-based functional connectivity.

Seed	Brain region	x	y	z	Ke	Peak t value	Seed’s system
**MPG > OOG**							
lBLA	Visual—Cuneus	10	− 80	18	329	4.76	Impulsive
lVLPFC	Brainstem	4	− 20	− 32	533	5.77	Reflective
	Thalamus	8	− 10	− 12	179	4.33	
	Thalamus	− 8	− 8	0	200	4.40	
	Insular cortex	− 32	− 20	8	117	4.38	
rDLPFC	Sensorimotor area	30	− 16	18	101	5.08	Reflective
lDLPFC	Middle temporal gyrus	− 46	− 50	16	126	4.35	Reflective
rPI	Fusiform gyrus	− 40	− 42	− 18	161	4.35	Interoceptive
	Heschl Gyrus	− 34	− 32	4	111	4.19	
lPI	Angular Gyrus	− 50	− 54	38	276	4.40	Interoceptive
	Middle Temporal area	− 56	− 40	− 8	479	5.42	
	Putamen	− 18	6	10	121	4.25	
	Dorsolateral cortex	− 30	30	18	162	4.20	
	Cerebellum	28	− 66	− 46	268	5.42	
	Inferior frontal area	− 46	22	14	135	4.70	
**MPG < OOG**							
No results							

Coordinates (x, y, z) are given in Montreal Neurological Institute atlas space (MNI). *Ke* cluster size in voxels; All the results have exceeded the minimum threshold p < 0.001 and number of voxels for each seed. *Seed’s system* system to which each seed corresponds according to the Triadic reflective–impulsive–interoceptive awareness model, *MPG* male perpetrators group, *OOG* other offenders group, *lBLA* left basolateral amygdala, *lVLPFC* left ventrolateral prefrontal cortex, *rDLPFC* right dorsolateral prefrontal cortex, *lDLPFC* left dorsolateral prefrontal cortex, *rPI* right posterior insula, *lPI* left posterior insula.

### Exploratory analyses: correlations between rsFC and behavioral variables in male perpetrators

Exploratory partial Pearson correlations were conducted to explore the associations between specific male perpetrators rsFC and executive functions and socioemotional processes.

Regarding executive functions, exploratory correlations showed a negative association between go/no go task and left PI. Regarding *socioemotional processes*, negative correlations were found between irrational thoughts about women and rsFC of left VLPFC and between cognitive reappraisal and rsFC of the right PI. Also, negative correlation was found between empathy and rsFC of the right PI and between difficulties in emotional regulation and rsFC of the right PI and right CMA. Pearson correlation coefficients of all these correlations ranged between − 0.408 and − 0.565. However, none of these results remain significant after Bonferroni correction for multiple comparisons. We additionally report all the correlational results at an uncorrected significance level of 0.05 in Supplementary File 4 and Supplementary Fig. 1. Finally, performance in executive functions and socioemotional measures is summarized in Supplementary File 5.

## Discussion

The main objective of this research was to study the resting-state functional connectivity underlying the process of social decision-making^12^ in male perpetrators convicted for an IPVAW crime and compare it to other offenders and non-offenders. Results showed that male perpetrators present different resting-state functional connectivity between brain areas related to impulsive, reflective and interoceptive systems.

Our main hypothesis proposed that male perpetrators would present disruptive resting-state connectivity between the reflective system and the impulsive and interoceptive brain systems in comparison to non-offenders and other offenders. Our results support this hypothesis. First, regarding *the reflective system*, male perpetrators presented higher functional connectivity between left ventrolateral prefrontal cortex seed and brainstem, middle temporal and limbic areas. Neuroimaging studies in male perpetrators suggest an inadequate prefrontal control in top-down regulatory control over excessive limbic activation^17^. Underlying the importance of adding the brainstem to this network due to its critical role in the integration of emotional stimulus^24^, this result might be pointing out a different resting-state functional connectivity in a vertical-integrative system composed by brainstem, limbic and cortical areas. Along the same line of reasoning, perpetrators showed higher functional connectivity between the reflective left dorsolateral prefrontal cortex seed and dorsal striatum in comparison to non-offenders. Dorsolateral prefrontal cortex is strongly related to the regulation of aggressive behavior^25^, while dorsal striatum has been found related to reinforcement of aggression^26^. Together, these two areas have an important role in down-regulation process. Indeed, literature demonstrated that in violent populations, the lateral prefrontal cortex exerts top-down control over subcortical areas such as striatum, in order to suppress output that lead to impulsive responses^27^. In addition, male perpetrators demonstrated lower resting-state functional connectivity between right ventrolateral prefrontal cortex seed and primary motor area, premotor area, and intraparietal sulcus. A recent study showed that a good cognitive and motor control requires the participation of a network comprising prefrontal areas, premotor and motor regions and that weaker connectivity between these areas is correlated with poorer inhibition^28^.

Regarding *the interoceptive brain system*, male perpetrators showed higher resting-state functional connectivity between the posterior insula seed and bilateral putamen. This connectivity has been studied in gaming disorders^29^ and in addiction^30^ demonstrating the strong relation between functional connectivity putamen-insula and the trait of impulsivity, a key component in intimate partner violence against women research. Posterior insula has also been found highly functionally connected with posterior areas of the default mode network in male perpetrators. This result is consistent with another study^19^ where they demonstrated that male perpetrators presented higher activation of the insula, angular gyrus and temporal areas when they viewed images of intimate partner violence. In fact, different studies have confirmed the functional connectivity between posterior insula and posterior areas of the default mode network proposing that self-reference and integrative/interoceptive processes are functionally connected^31^.

In relation to *the impulsive system*, male perpetrators presented lower resting-state functional connectivity between right centromedial amygdala seed and fusiform face area, and occipital superior area. These areas have been firmly found related to the emotion recognition process^32^. Increased functional coactivation between the fusiform gyrus and amygdala has been demonstrated when participants observed facial expressions of fear, disgust and happiness^33,34^. Also, male perpetrators demonstrated higher functional connectivity between left basolateral amygdala seed and left angular gyrus in comparison to non-offenders. The significant higher functional connectivity between these areas has not been assessed in previous studies in violent populations; however they have been found functionally or structurally altered in psychopathic/antisocial populations under moral decision making^16^.

Finally, in relation to the exploratory correlations, the results seem to suggest that male perpetrators' intrinsic connectivity might be more associated with socioemotional processes than with executive functions. Emotional regulation, empathy, emotion recognition and distorted thoughts, are part of what is now known as social cognition. This novel research field is defined as the study of all processes related to how people make sense of the world, events, other people and themselves, focusing on cognitive and affective processes and their corresponding biomarkers^35^. Over the last decade, research in male perpetrators has been exploring these processes as mediators^9,36–38^ in IPVAW^39,40^. Although the correlations do not remain significant after correction for multiple comparisons, these results are intended to support a novel research interest on social cognition in male perpetrators in order to promote further research and future replications (see Supplementary File 4).

The study results should be interpreted with a degree of caution due to several limitations: first, male perpetrators who presented a psychological disorder or had a history of substance abuse were excluded from the study in order to reduce the effect of confounders in the analysis. Second, even if seed-analysis has been one of the most used approaches in fMRI studies in violent populations^21^, the dependence on the selection of seed makes the analysis vulnerable to bias^41^. Third, due to the lack of specificity on the hypotheses regarding which connections and directions would be disrupted, the correlation analyses were exploratory in nature. Finally, since there are no previous resting-state studies in male perpetrators, the required sample size was calculated from a single fMRI study that uses a task-based approach comparing male perpetrators versus other offenders^19^. This power analysis could be biased and is likely using an inflated effect size, thus, the study may be underpowered. However, it should be noted that there is no neuroimaging study of male perpetrators with larger sample size.

Despite these limitations, this study provides the first evidence that resting-state functional connectivity is different in male perpetrators. These preliminary findings highlight that male perpetrators showed a specific resting-state functional connectivity between the reflective brain system and the impulsive and interoceptive brain systems. More concretely, relevant differences in rsFC have been found between prefrontal and limbic, middle temporal and brainstem areas supporting the idea of an altered top-down regulation^17^. These results reinforce previous neuroimaging studies with male perpetrators^19,20^ suggesting that this population is different to other offenders. As the authors of the Triadic Model^12^ reveal, each system can be more complex and connect other brain areas that have not been proposed by the model. Our results seem to support this complexity, emphasizing the specific connectivity between insula and default mode network, and between amygdala and basal ganglia areas, as brain areas specialized in interoception processes. Future research requires replicating the results with more robust analyses and, to take into account other key brain areas such as the default mode network and the cerebellum. Both networks have also been proved to be very relevant in the study of violence^9,42^. Finally, it is crucial to place emphasis on social cognition processes in IPVAW research. Future steps will be on validating neural models of social cognition in male perpetrators and studying if male perpetrators are not only different to other offenders but also different to other violent men.

This study brings new insights to the study of intimate partner violence. The results reveal that specific functional connectivity of male perpetrators might be related to social processes underlying IPVAW. Studying the brain functioning of male perpetrators will allow us to explore potential brain differences that may act as predictors of this specific violence^21^. For now, our results reinforce the need to integrate work on cognitive and affective control, empathy and emotional processing in intervention and prevention programs with male perpetrators^43,44^.

## Methods

### Participants

The study comprised 84 men (age ranged from 20 to 64 years) divided into 2 convicted groups and a group with no criminal records. The convicted groups involved 26 male perpetrators convicted of intimate-partner violence against women (MPG) and 29 men convicted of crimes other than IPVAW (other offenders group, OOG). The group of 29 non-convicted men was recruited from the general population (non-offenders group, NOG). The minimum sample-size was computed using the statistical tool G* Power^45^. According to a previous fMRI task-based study^19^ (that compared male perpetrators versus other offenders) they found an effect size Cohen’s d of 0.9, with an expected power of 0.8 and an assumed alpha-level of 0.05, the required sample size must be higher or equal to 25 per group. Authors are aware that this power analysis could be biased as the effect size might be inflated for resting-state studies, but this task based effect was selected in the absence of previous resting state studies in this specific population.

All the participants met the following inclusion criteria: men aged 18 years old or older. Further inclusion criteria were specific for each group. For the OOG group: being convicted of crimes other than intimate-partner violence (i.e., traffic violation, robbery, scams) and for the NOG group, not having prior criminal records. Finally, for the male perpetrators group, or MPG, they have to: be convicted of an intimate-partner violence crime, regulated by the law of IPVAW in Spain^46^. The exclusion criteria for all groups included: a history of serious antecedents of psychological and personality problems, neurological illness, illiteracy, head injury, history of drug abuse or dependence according to the DSM-IV^47^ and the presence of anomalies or any contraindications to MRI. To be certain that none of the participants from the non-offenders or the other offenders group had a history of IPVAW, those who obtained a score greater than or equal to 11 on the severity subscale of the Conflict Tactic Scale-2^23^ were excluded. This cut-off has been used in other studies with male perpetrators^8,9,19,20,48^. Characteristics of the sample are visualized in Table 1.

### Procedure

The experimental protocol was approved by the Research Ethics Committee of the University of Granada (number issued: 1000/CEIH/2019), and all methods were performed in accordance with the relevant guidelines and regulations. The convicted groups were recruited from the Center for Social Insertion (CSI) in Granada (Spain). Non-offenders were recruited via community advertisement (i.e., social media, academies).

Participants took part in two sessions. In the first session, the interview and tests were administered. During the second, all men underwent an fMRI scan. All participants were invited to collaborate voluntarily and anonymously and signed a written informed consent. They received 50 euros for participating in the study, and no penal benefit was obtained as compensation for the convicted groups.

### Materials

The Interview evaluating the risk of serious couple violence^49^ was used to assess socio-demographic information about the perpetrator and the victim, perpetrator’s profile, information about the relationship status, types of violence and vulnerability factors for the victim. Further questions regarding childhood violent experiences, the use of different substances and history of head injury were added to the interview.

The Spanish translation of The Conflict Tactic Scale-2 (CTS-2)^23^ was used to evaluate the severity of violence. This scale detects the frequency and the intensity of the violence (both physical and psychological) toward an intimate-partner. It also measures different conflict tactics used inside the relationship. In total, the scale comprises 39 items and 5 subscales (physical and psychological violence, sexual coercion, damages and negotiation).

A complete battery for the assessment of executive functions (updating process, response inhibition, decision making, and cognitive flexibility) and an evaluation of socioemotional processes using self-report measures and behavioral task (distorted thoughts about women and violence, empathy, emotion recognition, emotional regulation) and impulsivity were performed (all information in Supplementary File 1). A similar description of the methods can be found in a previously published study that is part of the same research project^20,48^.

### Statistical analyses

Demographic and behavioral data were analyzed using the Statistical Package for the Social Sciences, version 22 (SPSS; Chicago, IL, USA). ANOVA tests were used to assess potential differences between groups. Due to differences between groups in drug abuse, a variable of severity of drug use was calculated by the sum of the total of affirmative responses to the criteria of substance Use Disorders based on the DSM-IV, and the frequency of drug use ranging from 0 “never” to 6 “everyday”. This drug severity variable was used as a confounding factor in all analyses.

### Imaging data acquisition and preprocessing

The resting-state scan lasted 8 min and participants were instructed to lie still with their eyes closed and not move or fall asleep during the whole session. The data were collected on a 3.0 T MRI scanner (Siemens TRIO) located at the Mind, Brain and Behavior Research Center of the University of Granada (Spain). During the acquisition, a T2*-weighted echo-planar imaging (EPI) sequence was obtained through the following parameters: repetition time (TR) = 2.0 s; Echo time (TE) = 25 ms; Field of view (FOV) = 238 × 238 mm; Acquisition Matrix = 68 × 68; thirty-five 3.5 m axial slices, Voxel Size = 3.5 × 3.5 × 3.5 mm, 240 whole-brain volumes. A sagittal three-dimensional T1 weighted turbo-gradient-echo sequence (TR = 2300 ms; TE = 3.1 ms, FOV = 208; Voxel size = 0.8 × 0.8 × 0.8 mm, Number of slices = 208) was also obtained. Brain structural image allows the checking of gross anatomical abnormalities and was used during preprocessing to improve normalization of the functional data.

Brain images were preprocessed using the Functional Connectivity Toolbox (CONN^50^ running under Matlab R2017a (MatchWorks, Natick, MA). Preprocessing steps included: (1) realignment and slice-timing correction of the functional images (2) outlier detection using ART toolbox (3) denoising of confounding effects using the CompCor strategy, which included 5 principal components from the WM and CSF, 12 motion regressors and regressors of noise components (one for each identified outlier scan during the outlier identification step) and 2 regressors of effect of rest (3) segmentation of structural and functional data (4) coregistration of images using each participant’s anatomical scan (5) normalization of the functional images (6) reslice to a 2 mm voxel size in the Montreal Neurological Institute space and spatial smoothing using an 6-mm full-width-at-half-maximum (FWHM) isotropic Gaussian Kernel. Additional steps included denoising with a band-pass temporal filter (0.008–0.09 Hz) and linear detrending term. In order to avoid excessive motion and based on a previous study^51^, those participants that presented less than < 4 min of data were excluded. No participant was removed from the analysis for this reason.

### Functional connectivity analysis: seed-based analysis

According to the goal of the study, seeds’ selection was based on the Triadic Reflective–Impulsive–Interoceptive Awareness Model^12^. This model proposes three differentiated but dependent systems that play a crucial role in decision-making: the impulsive-amygdala-striatal system, the reflective-prefrontal system and the interoceptive-awareness insular system. As a result, in this first step, we selected 4 main areas from which final seeds would be generated: amygdala, prefrontal area, striatum and insula. According to cytoarchitectonic characteristics and previous functional connectivity studies, 15 seeds were generated from the main 4 areas in MNI stereotaxic space using the MarsBar toolbox for SPM12 (Table 4). More details about seed selection is presented in Supplementary File 2.

**Table 4:** Coordinates and radius for each selected seed.

Seeds		Shorthand term	Coordinates				Radius
**Impulsive system**			x	y		z	3.5 mm
Centromedial amygdala	R	rCMA	23	− 5		− 13	
	L	lCMA	− 19	− 5		− 15	
Basolateral amygdala	R	rBLA	29	− 3		− 23	
	L	lBLA	− 26	− 5		− 23	
Ventral striatum	R	rVS					
Ventral caudate			10	15		0	
Ventral nucleus accumbens			9	9		− 8	
Ventral striatum	L	lVS					
Ventral caudate			− 10	15		0	
Ventral nucleus accumbens			− 9	9		− 8	
**Reflective system**							6 mm
Medial prefrontal cortex	R	MPFC	7	44		− 4	
Dorsolateral prefrontal cortex	R	rDLPFC	45	36		16	
	L	lDLPFC	− 43	18		29	
Ventrolateral prefrontal cortex	R	rVLPFC	40	30		− 16	
	L	lVLPFC	− 33	33		− 10	
**Interoceptive system**							2 mm
Anterior insula	R	rAI	37	20		− 6	
	L	lAI	− 34	17		− 4	
Posterior insula	R	rPI	40	− 6		4	
	L	lPI	− 38	− 6		5	

Coordinates (x, y, z) are given in Montreal Neurological Institute atlas space (MNI).

Seed-based analyses were performed using the CONN toolbox v.17.f^50^ and SPM12 implemented in Matlab 2017a. First-level maps were estimated using a general linear model (GLM) regression analysis for each seed region, including its mean activity time courses with nuisance signals as regressors of no interest, including motion, white matter and CSF timeseries. Separate first-level analyses were computed for right and left regions of each seed and a high-pass filter with a 128 s cutoff period was used. Contrast images for each subject were entered in separate second-level one-way ANOVA models to investigate group differences in each seed’s connectivity. Age and drug severity were used as covariates of no interest.

Minimum threshold extents for all the fMRI analyses were estimated for multiple comparisons by Monte Carlo simulations using AlphaSim within the RESTplus V 1.2. The input parameters were a significant level of p < 0.001 and the actual smoothness of data after model estimation. A whole-brain mask (242 545 voxels; 2 × 2 × 2 mm) was used and imaging results were considered significant with a minimum cluster size ranging from 696 to 792 mm^3^ (87–99 voxels) depending on the seed (Supplementary File 3).

### Exploratory analyses: associations with executive functions, emotional and social processes in male perpetrators

As an exploratory aim, we conducted Partial Pearson correlations to examine the relationship between the specific resting-state functional connectivity of male perpetrators and the executive and socioemotional processes. Before performing the correlation analyses, we first extracted the mean value of each seed’s total functional connectivity (network) separately (i.e. the mean value of the connectivity network of the right PI seed). For that, we created a mask containing the total significant between-group functional connectivity differences of each seed.

Second, those behavioral variables that did not follow normal distribution, were normalized using the adequate formula in each case. More concretely, Distorted Thoughts about Women (IPDM) and the Use of Violence (IPDV) were normalized by applying the Napierian logarithm and cognitive reappraisal variable was normalized by squaring the original value.

Third, Partial Pearson correlations were performed between each seed’s rsFC networks and the executive functions and the socio-emotional variables, controlling for age and drug severity. The Statistical Package for the Social Sciences, version 22 (SPSS; Chicago, IL, USA) was used for these analyses based on a threshold at p < 0.05 and Bonferroni correction for multiple comparisons was performed.

## Supplementary Information

Supplementary Information.
