# Supplementary material for: Resting-state functional connectivity and socioemotional processes in male perpetrators of intimate partner violence against women
Source: Sci Rep. 2022 Jun 16;12:10090. doi: 10.1038/s41598-022-14181-2 (PMC9203491; doi:10.1038/s41598-022-14181-2)
Supplement: Supplementary file 1 — Supplementary Information. [file 41598_2022_14181_MOESM1_ESM.pdf]

Supplemental File 1. Executive functions and socioemotional assessment.

Emotional and social assessment

The Inventory of Distorted Thoughts about Women and Violence [1] was employed to measure *irrational thoughts*. This checklist consists of 29 binary items divided in two subscales: 13 items related to sexual roles and inferiority of women (IPDM), and 16 items about the use of violence as an acceptable method of conflict resolution (IPDV). Each affirmative response scores 1, therefore the higher the score, the greater the number of distorted thoughts within each subscale.

Empathy was measured using the Spanish version of the Interpersonal Reactivity Index [2]. The IRI consists of 28 items and 4 subscales (perspective taking, fantasy, empathic concern and personal distress) and uses a 5-point Likert scale ranging from 1 (does not describe me well) to 5 (describes me well).

For the evaluation of *emotion recognition,* we selected the Spanish translation of the revised version of the Eyes Test [3]. It consists of 36 photographs of eyes and in each item participants are instructed to choose among four descriptors based on what they think the person in the photography is feeling. Each item is scored as correct or incorrect. Consequently, the higher the score, the greater is the ability to recognize emotions.

In order to evaluate *emotion regulation*, we used two different scales:

The Difficulties in Emotional Regulation Scale (DERS) in it Spanish version [4]. This checklist evaluates different aspects of the emotional regulation through 28 items which are answered on a 5-point Likert Scale ranging from 1 “almost never” to 5 “almost always”. Five subscales are assessed: lack of awareness, lack of emotional clarity, non-acceptance of emotional responses, difficulties in goal directed behavior and emotional dyscontrol. Consequently, the higher the score, the greater are the difficulties in emotional regulation.

The Spanish version of the Emotional Regulation Questionnaire [5]. It is a 10-item scale designed to measure the capacity to regulate emotions through two processes: cognitive reappraisal (6 items) and expressive suppression (4 items). Participants answer on a 7-point Likert scale ranging from 1 “strongly disagree” to 7 “strongly agree”.

For our subsequent correlational analyses, we used the total score of each of the questionnaires explained above except for the emotional regulation questionnaire (ERQ), where cognitive reappraisal and expressive suppression were considered as two different dependent variables.

Executive functions assessment

Four components of executive functioning were measured based in a previous study [6]. These four components were selected based on prior literature, where differences in male perpetrator were found [7].

Updating process: the letter-number sequencing [8] was used. Participants were asked to repeat a combination of an increasingly longer list of numbers and letters by organizing the numbers in ascending order and the letters in alphabetical order. The total number of correct answers was used as an index of updating ability.

Response inhibition: a go/no go task was employed using a tablet-based task of 100 trials divided into 4 blocks. In the first 50 trials (1° and 2° blocks), participants were asked to press a key as quickly as possible when the GO stimulus was presented (a black figure of a bear) and to inhibit the response for the NO-GO trials (a black figure of a dolphin). Then, participants hear a bell and have to change their response from the GO to the NO-GO stimulus for the next 50 trials (3° and 4° blocks). Thus, participants were asked to press the key when the NO-GO stimulus appeared and to inhibit the answer for the GO trials. The interstimulus interval (ISI) was established in 1.000ms, and each stimulus was presented for 500ms. The score resulting from the subtraction of the 3° block and the 2° block was used as an indirect measure of response inhibition.

Decision making: a computerized version of the Iowa Gambling Task [9] was used. This task involved four decks of cards (A, B, C and D). Participants were told to choose one card at a time from one of the

4 decks and after each choice, they received an economic reward and in some cases they also received an economic punishment. A and B decks were disadvantageous because even if they produced higher immediate gains they also resulted in higher punishment points. C and D decks were advantageous, because they resulted in modest rewards but lower punishment. In the long run, choosing from the advantageous decks would yield more money and less punishment. The variable was first calculated by subtracting the number of disadvantageous choices from the advantageous choices for each block (5 blocks of 20 trials). Then, a sum of all blocks' results was computed and used as an indirect decision-making proxy.

Cognitive flexibility: due to technical problems, instead of using the Trail Making Test as in the previous study [6] with male perpetrators, we selected the arrow-version of the Spatial Stroop task [10]. Participants were asked to respond to the left/right direction of an arrow regardless of its left/right position on a tablet screen. On congruent trials, the direction in which the arrow pointed was consistent with its location (right pointing arrow located in the right side, or left pointing arrow located in the left side). On incongruent trials, the arrow’s direction was opposite to its location (right pointing arrow located in the left side or left pointing arrow located in the right side). The attentional cost variable was taken as an indirect measure of cognitive flexibility. It was calculated by subtracting the reaction time of the change-trials (when a trial is preceded by a different trial) from the first-repetition-trials (when a trial is preceded by the same trial).

Impulsivity: for this purpose, we selected the Spanish short version of the Impulsive Behavior Scale [11]. It consists of 20 items that measure 5 impulsivity traits: negative, urgency, lack of premeditation, lack of perseverance, sensation of seeking and positive urgency. The items are scored on a 4-point Likert Scale ranging from 1 “strongly agree” to 4 “strongly disagree”. The higher the score, the higher the impulsivity. We used the total score of the scale as a measure of impulsivity trait for the correlational analysis.

Supplemental File 2. Seed Selection and Generation

The selection of the seeds was built on the Triadic Reflective-Impulsive-Interoceptive Awareness Model of Turel and Bechara [12]. This neurocognitive model was created to study the basis of problematic behaviors. It is based on the premise that there are three differentiated but dependent systems that play a crucial role in decision-making: The Impulsive amygdala-striatal brain system which mediates the generation of impulses to act headfirst in order to obtain incentive rewards and it is also important to form habituated and automatic behaviors [13]. Second, the Reflective prefrontal brain system, which is thought to be involved in executive functions such as working toward a defined goal, uses conscious reflections, manages predictions and expectation of outcomes and is involved in social control [14]. Finally, the Interoceptive-awareness insular brain system is implicated in perceiving, processing and representing afferent internal bodily signals [15], essential for a functional social behavior. As a result, in this first step, we selected 4 main areas from which final seeds would be generated: amygdala, prefrontal area and striatum and insula.

Within the impulsive system, amygdala was divided into: centromedial amygdala (rCMA and lCMA) and basolateral amygdala (rBLA and lBLA) regions according to its cytoarchitectonic characteristics ([16]. We chose from the striatum, the ventral striatum (VS). Both rVS and lVS masks involved the lateralized ventral caudate (VSi) and nucleus accumbens (VSs), directly related to the rewarding process. Local maxima coordinates were obtained from the article Functional Connectivity of Human Striatum: A Resting State fMRI Study [17]. The amygdala-striatal seeds were located using a 3.5-mm-radius spheres. Within the reflective system, the prefrontal cortex was divided into: medial prefrontal cortex (MPFC), bilateral dorsolateral prefrontal cortex (rDLPFC and lDLPFC) and bilateral ventrolateral prefrontal cortex (rVLPFC and lVLPFC) [18, 19]. The mean activity of each prefrontal seed was extracted from a 6-mm-radius sphere. Finally, within the interoceptive-awareness system, theinsula was divided in left and right anterior (AI) and posterior insula (PI), based on the assumption thateach one belongs to dissociable resting-state networks [20]. Insula' seeds were located using a 2-mm- radius spheres. In total, 15 seed were generated in MNI stereotaxic space using the MarsBar toolbox forSPM12 (http://marsbar.sourceforge.net).

Supplemental File 3. Selected seeds and minimum cluster size for correction.

SEEDS	FWHM (mm)	Minimum cluster size
Impulsive system		
rBLA	8.6	87
lBLA	8.6	91
rCMA	8.7	97
lCMA	8.8	94
rVS	8.7	92
lVS	8.7	96
Reflective system		
MPFC	9	99
rDLPFC	8.96	97
lDLPFC	9	99
rVLPFC	8.9	93
lVLPFC	9.03	98
Interoceptive system		
rAI	8.7	94
lAI	8.9	92
rPI	8.7	99
lPI	8.9	93

FWHM = Full width at half maximum. Minimum cluster size were estimated for multiple comparisons by Monte Carlo simulations using AlphaSim within RESTplus toolbox. rBLA = right basolateral amygdala; lBLA = left basolateral amygdala; rCMA = right centromedial amygdala; lCMA = left centromedial amygdala; rVS = right ventral striatum; lVS = left ventral; striatum; MPFC = medial prefrontal cortex; rDLPFC = right dorsolateral prefrontal cortex; lDLPFC = left dorsolateral prefrontal cortex; rVLPFC = right ventrolateral prefrontal cortex; lVLPFC = left ventrolateral prefrontal cortex; rAI = right anterior insula; lAI = left anterio insula; rPI = right posterior insula; lPI = left posterior insula.

Supplemental File 4. Exploratory correlations without correction for multiple comparisons.

Exploratory partial Pearson correlations were conducted to explore the associations between specific male perpetrators rsFC and executive functions and socioemotional processes. To this end, the mean value of each seed found significantly different in MPG in comparison to NOG and OOG were correlated with the selected behavioral measures

- **Executive functions.** In MPG, the rsFC between left posterior insula and posterior default mode network (pDMN), prefrontal and cerebellum areas correlated negatively with the go/nogo task (r = -.427, p = .042). No other significant correlation was found between the executive function measures and resting-state functional connectivity.
- **Socioemotional processes.** In MPG, the rsFC between lVLPFC seed and brainstem, hippocampus and middle temporal area correlated negatively with irrational thoughts related to sexual roles and inferiority of women (r = -516, p = .010). The rsFC between rPI seed, fusiform gyrus and Heschl gyrus correlated negatively with empathy (r = -.499, p = .013), cognitive reappraisal (r = -.447, p = .037) and difficulties in emotion regulation (r = -.565, p = .014). Also, the rsFC between rPI seed and bilateral putamen correlated negatively with empathy (r = -.408, p = .048). Finally, rsFC between right rCMA seed and intraparietal area, occipital and fusiform gyrus correlated negatively with difficulties in emotion regulation (r = -.483, p = .042).

* Even though these results appear to be contradictory, it might be explained by the model upon which each scale is based. Concretely, DERS scale [21] is based on a clinical-contextual model of emotional regulation and therefore focuses on presumed trait-level abilities. By contrast, ERQ questionnaire [22] was created from an affective science-based framework and attended to processes related to emotional regulation [23].

Supplemental Figure 1. Significant Pearson partial correlation between seeds' functional connectivity and executive functions and socioemotional processes in MPG

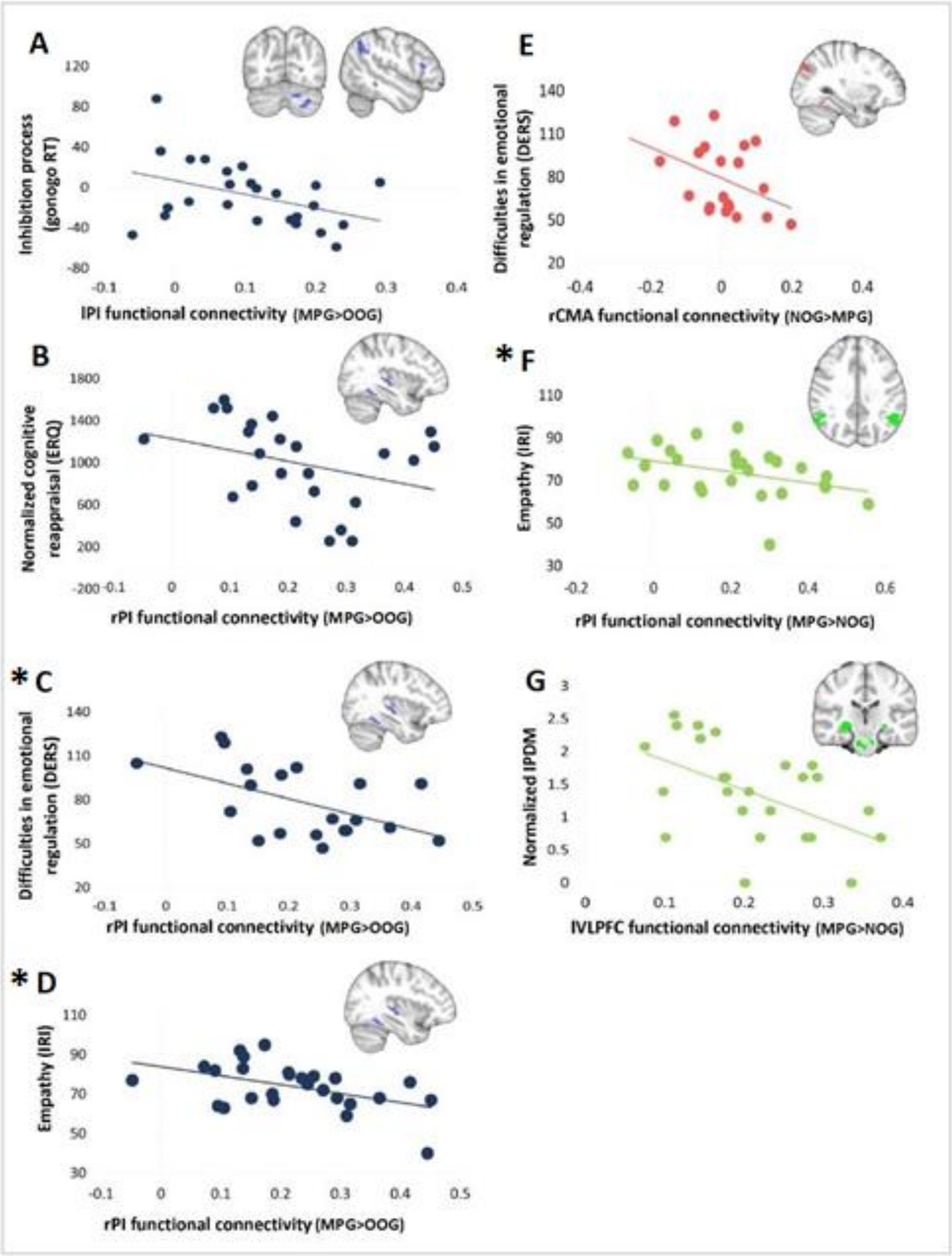

Supplemental File 5. Performance of MPG, OOG and NOG in executive functions, and socioemotional measures

VARIABLES		MPG		OOG		NOG
Executive functions	**N**		**N**		**N**	
Lns	25	7.24 (3.09)	29	7.45 (3.29)	29	9.10 (2.67)
IGT	26	-1.31 (17.49)	29	-3.31 (20.66)	29	8.62 (22.79)
Gonogo RT	25	-7.64 (32.44)	28	-14.20 (38.86)	29	-12.72 (27.97)
Atentional cost	26	-78.14 (66.06)	29	-93.89 (51.17)	28	-111.12 (54.84)
Socioemotional variables						
Empathy (IRI)	26	73.85 (11.48)	24	83.46 (9.89)	27	82.89 (11.15)
Normalized cognitive reappraisal (ERQ)	24	997.46 (406.00)	28	923.68 (411.64)	29	914.79 (427.31)
Expressive suppression (ERQ)	24	18.25 (6.71)	28	16.00 (6.45)	29	15.28 (5.09)
Difficulties in emotion regulation (DERS)	20	78.35 (23.98)	24	66.21 (21.57)	25	69.32 (18.00)
Normalized IPDV	26	1.21 (0.60)	29	1.39 (0.59)	29	0.88 (0.52)
Normalized IPDM	26	1.37 (0.72)	29	1.56 (0.68)	29	1.19 (0.60)
Eyes Test	16	17.02 (4.66)	15	19.40 (4.35)	17	20.71 (4.31)
UPPS	20	42.75 (8.932)	23	45.00 (9.977)	25	44.32 (7.609)

All values are mean (± SD). MPG = male perpetrators group, OOG = other offenders group; NOG = non-offenders group. N = sample size.

Lns = letters and numbers correct answers (updating process); answers; IGT = Iowa Gambling Task (decision-making process); Gonogo RT = Gonogo reaction time (response inhibition) measured in miliseconds; IRI = Interpersonal Reactivity Index (Empathy); Cognitive reappraisal of the Emotional Regulation Questionnaire (ERQ); Expressive suppression of the Emotional Regulation Questionnaire (ERQ); DERS = Difficulties in Emotion Regulation Scale; IPDV = Inventory of distorted Thoughts about the Use of Violence; IPDM = Inventory of distorted Thoughts about Women; Eyes test (emotion recognition); UPPS = Impulsive Behavior Scale (impulsivity).

References

1. Echeburua, E. & Fernandez-Montalvo, J. Cognitive-Behavioural Treatment of Violent Men in the Home: A Pilot Study. Analysis and Modification of Behaviour 23, 355–384 (1997).
2. Pérez-Albéniz, A., De Paúl, J., Etxeberría, J., Montes, M. P. & Torres, E. Adaptación de interpersonal reactivity index (IRI) al español. (2003).
3. Fernández-Abascal, E. G., Cabello, R., Fernández-Berrocal, P. & Baron-Cohen, S. Test-retest reliability of the 'Reading the Mind in the Eyes' test: a one-year follow-up study. Molecular autism 4, 1–6 (2013).
4. Hervás, G. & Jódar, R. Adaptación al castellano de la Escala de Dificultades en la Regulación Emocional. Clínica y salud 19, 139–156 (2008).
5. Cabello, R., Salguero, J. M., Fernández-Berrocal, P. & Gross, J. J. A Spanish adaptation of the emotion regulation questionnaire. European Journal of Psychological Assessment (2013).
6. Bueso-Izquierdo, N., Hidalgo-Ruzzante, N., Daugherty, J. C., Burneo-Garcés, C. & Pérez-García, M. Differences in executive function between batterers and other criminals. Journal of forensic psychology practice 16, 321–335 (2016).
7. Horne, K., Henshall, K. & Golden, C. Intimate partner violence and deficits in executive function. Aggression and violent behavior 101412, (2020).
8. Amador, J. A. Escala de inteligencia de Wechsler para adultos-IV (WAIS-IV. (2013).
9. Bechara, A., Damasio, H., Tranel, D. & Damasio, A. R. The Iowa Gambling Task and the somatic marker hypothesis: some questions and answers. Trends in cognitive sciences 9, 159–162 (2005).
10. Luo, C., Lupiáñez, J., Funes, M. J. & Fu, X. Reduction of the spatial Stroop effect by peripheral cueing as a function of the presence/absence of placeholders. PLoS One 8, 69456 (2013).
11. Cándido, A., Orduña, E., Perales, J. C., Verdejo-García, A. & Billieux, J. Validation of a short Spanish version of the UPPS-P impulsive behaviour scale. Trastornos adictivos 14, 73–78 (2012).

12. Turel, O. & Bechara, A. A triadic reflective-impulsive-interoceptive awareness model of general and impulsive information system use: Behavioral tests of neuro-cognitive theory. Frontiers in Psychology 7, 601 (2016).
13. Yin, H. H. & Knowlton, B. J. Addiction and learning in the brain. in Handbook of Implicit Cognition and Addiction 167–183 (2006).
14. Siddiqui, S. V., Chatterjee, U., Kumar, D., Siddiqui, A. & Goyal, N. Neuropsychology of prefrontal cortex. Indian Journal of Psychiatry 50, 202 (2008).
15. Critchley, H. D. & Garfinkel, S. N. Interoception and emotion. Current Opinion in Psychology 17, 7–14 (2017).
16. Baur, V., Hänggi, J., Langer, N. & Jäncke, L. Resting-state functional and structural connectivity within an insula–amygdala route specifically index state and trait anxiety. Biological Psychiatry 73, 85–92 (2013).
17. Di Martino, A et al. Functional connectivity of human striatum: A resting state FMRI study. Cerebral Cortex 18, 2735–2747 (2008).
18. MacDonald, A. W., Cohen, J. D., Stenger, V. A. & Carter, C. S. Dissociating the role of the dorsolateral prefrontal and anterior cingulate cortex in cognitive control. Science 288, 1835–1838 (2000).
19. Ridderinkhof, K. R., Van Den Wildenberg, W. P., Segalowitz, S. J. & Carter, C. S. Neurocognitive mechanisms of cognitive control: The role of prefrontal cortex in action selection, response inhibition, performance monitoring, and reward-based learning. Brain and Cognition 56, 129–140 (2004).
20. Cauda, F. et al. Functional connectivity of the insula in the resting brain. Neuroimage 55, 8–23 (2011).
21. Gratz, K. L. & Roemer, L. Multidimensional assessment of emotion regulation and dysregulation: Development, factor structure, and initial validation of the difficulties in emotion regulation scale. Journal of psychopathology and behavioral assessment **26**, 41–54 (2004).
22. Gross, J. J. & John, O. P. Individual differences in two emotion regulation processes: implications for affect, relationships, and well-being. Journal of personality and social psychology **85**, 348 (2003).

23. Hallion, L. S., Steinman, S. A., Tolin, D. F. & Diefenbach, G. J. Psychometric properties of the Difficulties in Emotion Regulation Scale (DERS) and its short forms in adults with emotional disorders. Frontiers in psychology **9**, 539 (2018).
